# Supplementary material for: Cross‐Species Prediction of Transcription Factor Binding by Adversarial Training of a Novel Nucleotide‐Level Deep Neural Network
Source: Adv Sci (Weinh). 2024 Jul 30;11(36):2405685. doi: 10.1002/advs.202405685 (PMC11423150; doi:10.1002/advs.202405685)
Supplement: Supplementary file 1 — Supporting Information [file ADVS-11-2405685-s001.pdf]

ADVANCED SCIENCE
Open Access

Supporting Information

for *Adv. Sci.*, DOI 10.1002/advs.202405685

Cross-Species Prediction of Transcription Factor Binding by Adversarial Training of a Novel Nucleotide-Level Deep Neural Network

*Qinhu Zhang, Siguo Wang, Zhipeng Li, Yijie Pan and De-Shuang Huang**

Supporting Information for:

Cross-species prediction of transcription factor binding by adversarial training of a novel nucleotide-level deep neural network

Qinhu Zhang[1,2,3], Siguo Wang[1], Zhipeng Li[1], Yijie Pan[1], and De-Shuang Huang[1,4*]
[1] Ningbo Institute of Digital Twin, Eastern Institute of Technology, Ningbo, 315201, China.
[2] Division of Life Sciences and Medicine, University of Science and Technology of China, Hefei, 230021, China
[3] Big Data and Intelligent Computing Research Center, Guangxi Academy of Science, Nanning, 530007, China.
[4] Institute for Regenerative Medicine, Shanghai East Hospital, Tongji University, Shanghai, 200092, China.
* To whom correspondence should be addressed. Email: dshuang@eitech.edu.cn

Supplementary Notes

Supplementary Note 1: Constructing no-SINE models on both human and mouse species

Cochran et al.[1] have systematically investigated the effect of repeat elements (specifically *Alu* elements, a type of SINE) on the performance of cross-species TF binding prediction, concluding that reduced cross-species predictive performance can directly be attributed to one type of species-specific repeats. Inspired by this work, we constructed no-SINE models on both human and mouse species following these steps: (i) we downloaded human and mouse's repeat elements from UCSC, respectively (http://hgdownload.cse.ucsc.edu/goldenPath/hg38/database/rmsk.txt.gz; http://hgdownload.cse.ucsc.edu/goldenPath/mm10/database/rmsk.txt.gz); (ii) we picked out SINE elements belonging to *Alu* family from the two files, (iii) we processed ChIP-seq data for human and mouse using the same way described in the main text; (iv) based on NLDNN, we trained no-SINE models on both human and mouse species by filtering out any training windows that intersect with *Alu* elements, and then evaluated them using all test windows which includes *Alu* elements. The PR-AUC and Pearson correlation were used in the evaluation process.

Supplementary Note 2: Designing experiments for investigating the effect of the sequence context features across species

As mentioned in the main text, human and mouse motifs are highly similar, e.g., 98% similarity for CTCF motif, meaning that their TF binding sites are also similar. However, a recent study has proved that the sequence context features (flanking sequences) of TF binding sites are still predictive of TF binding[2]. In this study, we assume that this factor may influence the performance of cross-species TF binding prediction. To investigate the effect of the sequence context features across species, we designed an experiment by relying on the sequence context features only to discriminate cross-species CTCF binding sites. Specifically, (i) we selected ChIP-seq data for TF CTCF from three cell types (lines); (ii) we processed ChIP-seq data for human and mouse using the same way described in the main text except that taking one species as the positive set while another species as the negative set, and then filtered out any windows that intersect with *Alu* elements to eliminate the impact of repeat elements; (iii) we employed FIMO[3] to search for CTCF motif instances of high significance (p-value < 1e-4) from TF binding peaks and then masked these instances from binding peaks by setting their encoded vectors as 0. Here we only masked CTCF-like binding sites of high significance from the training set without masking the test set; (iv) we trained NLDNN to discriminate two species' binding peaks and evaluated it using the PR-AUC and ROC-AUC metrics.

Supplementary Note 3: Investigating the predictive performance of NLDNN on ChIP-nexus binding data

Data preparation

To investigate the predictive performance of NLDNN on ChIP-nexus binding data, i) we downloaded four Mouse ChIP-nexus data (Klf4, Nanog, Oct4, and Sox2) from the literature[4]; ii) we used all peaks as the positive set and cut them into sequences of 1000bp; iii) we extracted the upstreaming regions 3000bp away from these peaks as the negative set and similarly cut them into sequences of

1000bp; iv) we transformed each sequence into a matrix of 4*1000 where '4' represents four types of nucleotide {A, C, G, T}; v) since ChIP-nexus technology will produce positive-strand and negative-strand coverage signals, so each positive sequence has two coverage signals. Instead, the two coverage signals of each negative sequence are both set to zero.

Chromosomes 1, 18, and 8 were held out from all training data (excluding Chromosome Y) where Chromosome 8 was used as the validation set for hyper-parameter tuning while Chromosome 1 and 18 were used as the test set.

NLDNN training

Since each sequence has two coverage signals, so we adopted a combination of loss on positive and negative strands, $\text{Loss} = \text{Loss}_{positive} + \text{Loss}_{negative}$. Here, we tried two types of loss functions, including Mean Squared Error (MSE) and Poission Loss.

We employed a 'warm-up' strategy to reduce the impact of parameter initialization on NLDNN. Specifically, we first warmed up NLDNN by running a few randomly initialized models and selecting the best-performing model in terms of validation accuracy. Then, NLDNN was initialized by the best-performing model and unceasingly trained with a batch size of 500 using ADAM with default parameters. The number of training epochs was set to 60, and the initial learning rate was set to 0.001 and decayed by a factor of 0.9 every 10 epochs.

After training, we can use the trained model to predict the ChIP-nexus profiles of Oct4, Sox2, Nanog, and Klf4.

The competing method BPNet

The literature[4] has provided the original BPNet model jointly trained using the four Mouse ChIP-nexus data (Klf4, Nanog, Oct4, and Sox2). The original BPNet model is available on Kipoi under the name BPNet-OSKN (http://kipoi.org/models/BPNet-OSKN/). After installation, we can use it to predict the ChIP-nexus profiles of Oct4, Sox2, Nanog, and Klf4.

Evaluation

To evaluate the predictive performance of NLDNN and BPNet, PR-AUC (Area Under the Precision-Recall Curve) and Pearson correlation were adopted to verify their classification and fitting performance. Specifically, the maximums of predicted and true coverage values were used to calculate the PR-AUC and Pearson correlation.

Supplementary Note 4: Investigating the cross-species predictive performance of NLDNN-AT on ChIP-seq binding data associated with Drosophila Melanogaster

Data preparation

To investigate the the cross-species predictive performance of NLDNN, i) we downloaded two ChIP-seq data associated with Drosophila Melanogaster (dm6) from ENCODE, and used the same procedure described in the main text to process these data; ii) we chose 'Human' as the source species labeled 1 and 'Drosophila Melanogaster' as the target species labeled 0. Given that the source species is observed and binding sequences are informative of TF binding, binding sequences from the source species were therefore used as the source data. On the contrary, since the target species is

unobserved, arbitrary sequences from the target species were used as the target data.

NLDNN-AT training

The training process of NLDNN-AT was performed following the same procedure described in the main text. Briefly, we first trained NLDNN using ChIP-seq binding data associated with dm6, and then used the dual-path framework to fine-tune NLDNN by adversarial training.

Evaluation

To evaluate the cross-species predictive performance of NLDNN-AT, PR-AUC was used to evaluate the classification performance, and Pearson correlation was used to evaluate the fitting performance.

Supplementary Note 5: Applying the trained Enformer to predict TF binding

Although Enformer[5] was designed for predicting gene expression from long-range sequences, it adopted a multi-task framework to predict gene expression and chromatin states, which are closely related to TF binding activity. Therefore, we can apply the trained Enformer to predict TF binding.

Data preparation for inference

For adapting to Enformer, i) we used all peaks as the positive set and cut them into sequences of 393216bp; iii) we extracted the upstreaming regions 3000bp away from these peaks as the negative set and similarly cut them into sequences of 393216bp; iv) we transformed each sequence into a matrix of 4*393216 where '4' represents four types of nucleotide {A, C, G, T}.

Since we just performed model inference, sequences from Chromosomes 1 and 18 (the test set) were only used.

Model inference

Pre-trained Enformer model was downloaded from https://github.com/google-deepmind/deepmind-research/tree/master/enformer. The details of inference can refer to the section of Running Inference on Github. More specifically, i) the Enformer model took a sequence of length 393216 as input and output predictions corresponding to 128 base pair windows of the center 114,688 base pairs. The predictions have two branches, one branch for human predictions (shape: 896×5313) and another for mouse predictions (shape: 896×1643); ii) we searched for the corresponding cell type and TF name among human or mouse predictions, and then extracted and aggregated all matched predictions; iii), we took the maximum prediction for 6 bins surrounding the central bin as the final prediction.

Evaluation

To evaluate the predictive performance of Enformer, PR-AUC was used to evaluate the classification performance, and Pearson correlation was used to evaluate the fitting performance.

Supplementary Note 6: Conducting ablation experiments to investigate the contributions of the main modules to NLDNN

According to the description of NLDNN, except for the basic architecture (e.g., three convolutional and up-sample blocks), it has three main modules that contribute to the prediction performance,

including bi-directional GRU (Gated Recurrent Unit), SPPM (Simple Pyramid Pooling Module), and RRB (Refinement Residual Block). To investigate the contribution of each module, we conducted ablation experiments by iteratively removing each module from NLDNN. The predictive performance of each module was evaluated using 7 Erythroid-specific ChiP-seq binding datasets. As shown in Supplementary Fig.13d, the overall predictive performance of NLDNN is better than that of incomplete modules, demonstrating that the three modules all have a positive effect on the prediction performance of NLDNN, in particular, the contribution of RRB is the most notable.

References

1. Cochran, Kelly, et al. "Domain-adaptive neural networks improve cross-species prediction of transcription factor binding." Genome research 32.3 (2022): 512-523.
2. Zheng, An, et al. "Deep neural networks identify sequence context features predictive of transcription factor binding." Nature machine intelligence 3.2 (2021): 172-180.
3. Grant, Charles E., Timothy L. Bailey, and William Stafford Noble. "FIMO: scanning for occurrences of a given motif." Bioinformatics 27.7 (2011): 1017-1018.
4. Avsec, Žiga, et al. "Base-resolution models of transcription-factor binding reveal soft motif syntax." Nature genetics 53.3 (2021): 354-366.
5. Avsec, Žiga, et al. "Effective gene expression prediction from sequence by integrating long-range interactions." Nature methods 18.10 (2021): 1196-1203.

Supplementary Figures

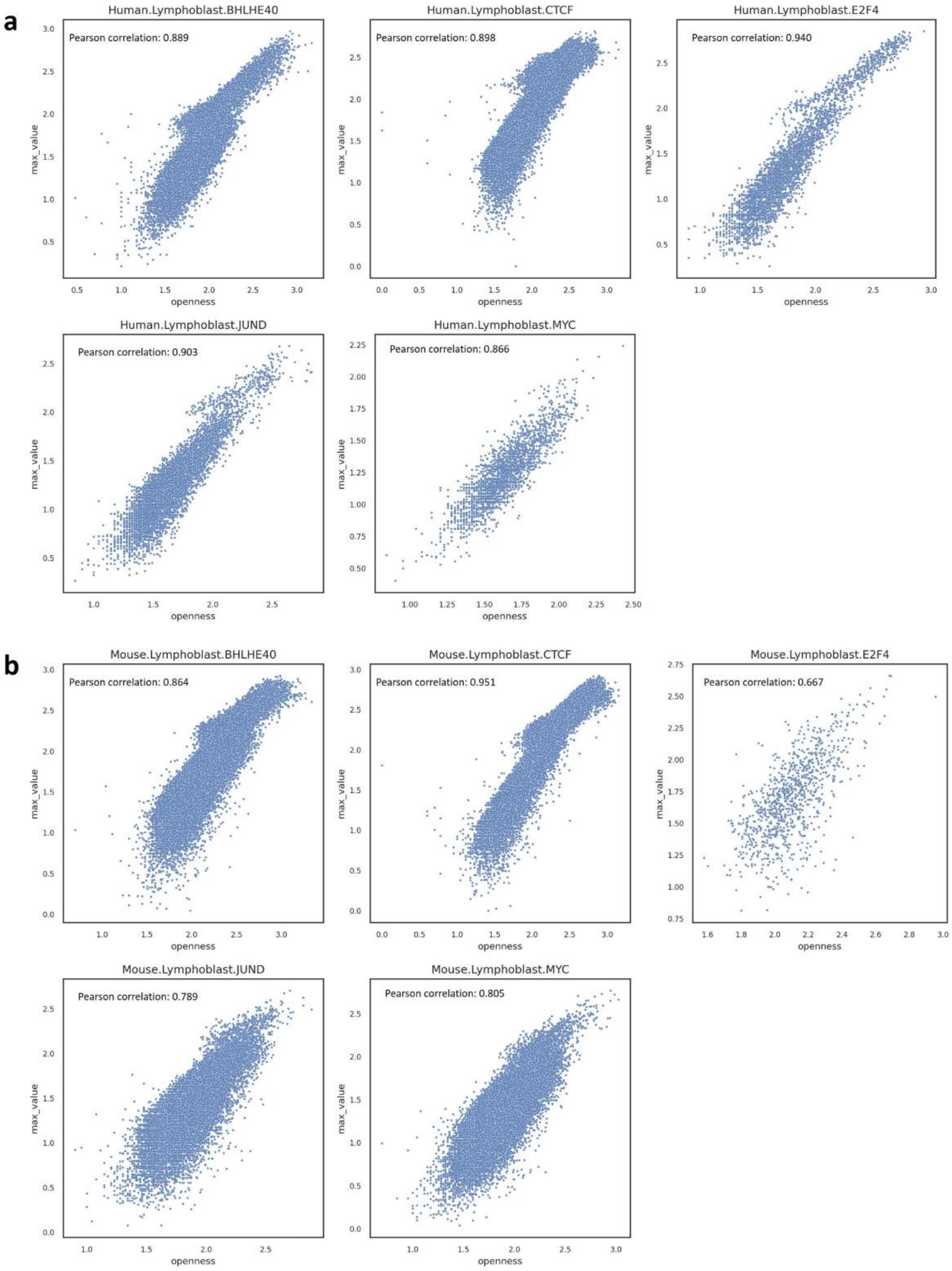

Figure S1. Relationship between the maximum of coverage values and the strength of TF binding. (a) Pearson correlation between the maximums of coverage values and the strength of TF binding for five human TF factors. (b) Pearson correlation between the maximums of coverage values and the strength of TF binding for five mouse TF factors.

a

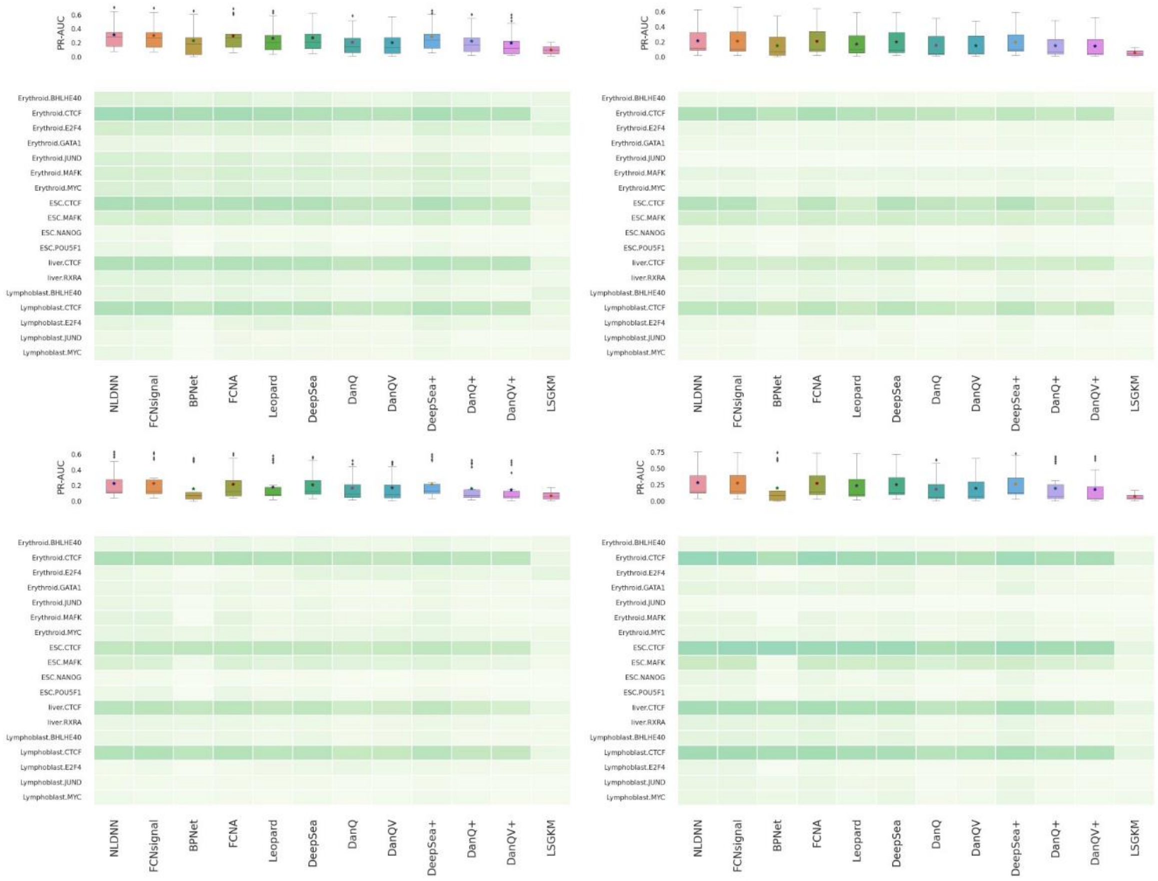
PR-AUC
Erythroid.BHLHE40
Erythroid.CTCF
Erythroid.E2F4
Erythroid.GATA1
Erythroid.JUND
Erythroid.MAFK
Erythroid.MYC
ESC.CTCF
ESC.MAFK
ESC.NANOG
ESC.POU5F1
liver.CTCF
liver.RXRA
Lymphoblast.BHLHE40
Lymphoblast.CTCF
Lymphoblast.E2F4
Lymphoblast.JUND
Lymphoblast.MYC
NLDNN
FCNsignal
BPNet
FCNA
Leopard
DeepSea
DanQ
DanQV
DeepSea+
DanQ+
DanQV+
LSGKM

b

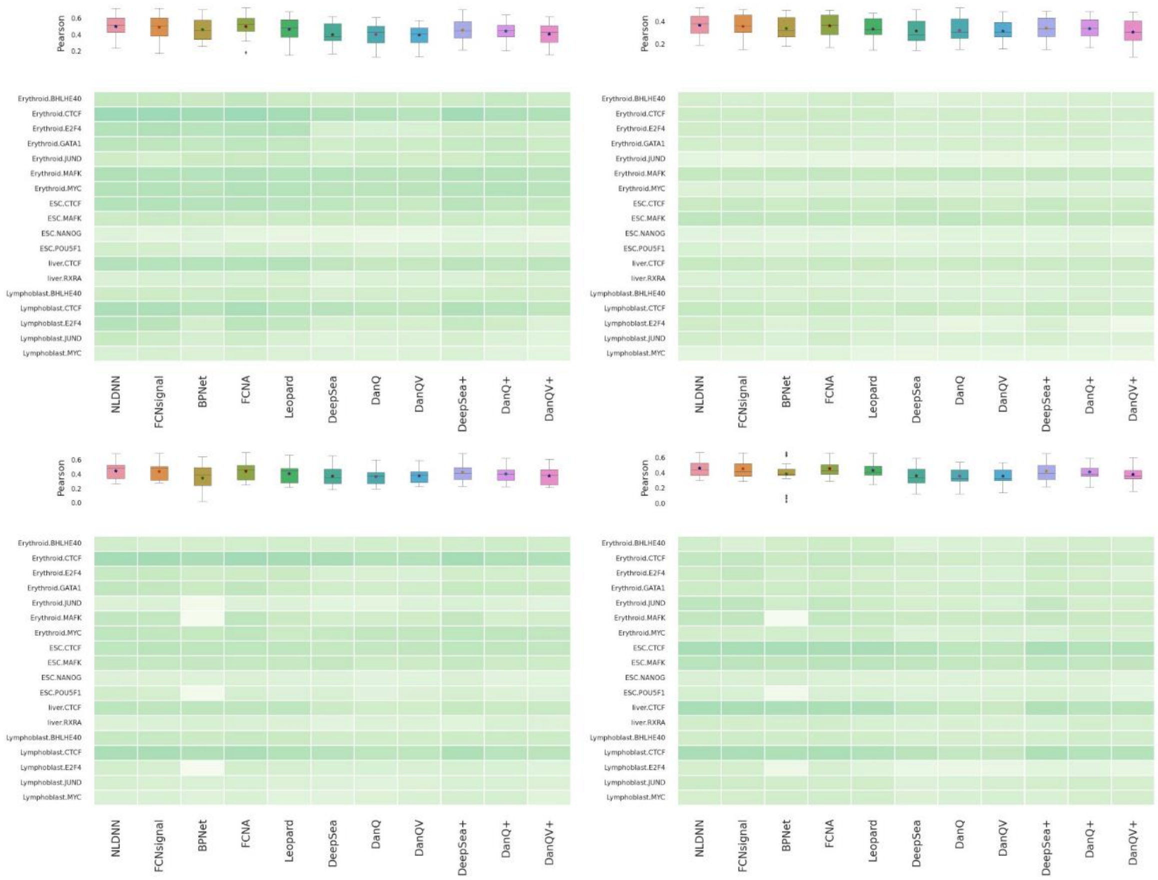
Pearson
Erythroid.BHLHE40
Erythroid.CTCF
Erythroid.E2F4
Erythroid.GATA1
Erythroid.JUND
Erythroid.MAFK
Erythroid.MYC
ESC.CTCF
ESC.MAFK
ESC.NANOG
ESC.POU5F1
liver.CTCF
liver.RXRA
Lymphoblast.BHLHE40
Lymphoblast.CTCF
Lymphoblast.E2F4
Lymphoblast.JUND
Lymphoblast.MYC
NLDNN
FCNsignal
BPNet
FCNA
Leopard
DeepSea
DanQ
DanQV
DeepSea+
DanQ+
DanQV+

Figure S2. Overall predictive performance of all models for predicting within- and cross-species TF binding. (a) Within- and cross-species classification performance of all models (PR-AUC). (b) Within- and cross-species fitting performance of all models (Pearson correlation)

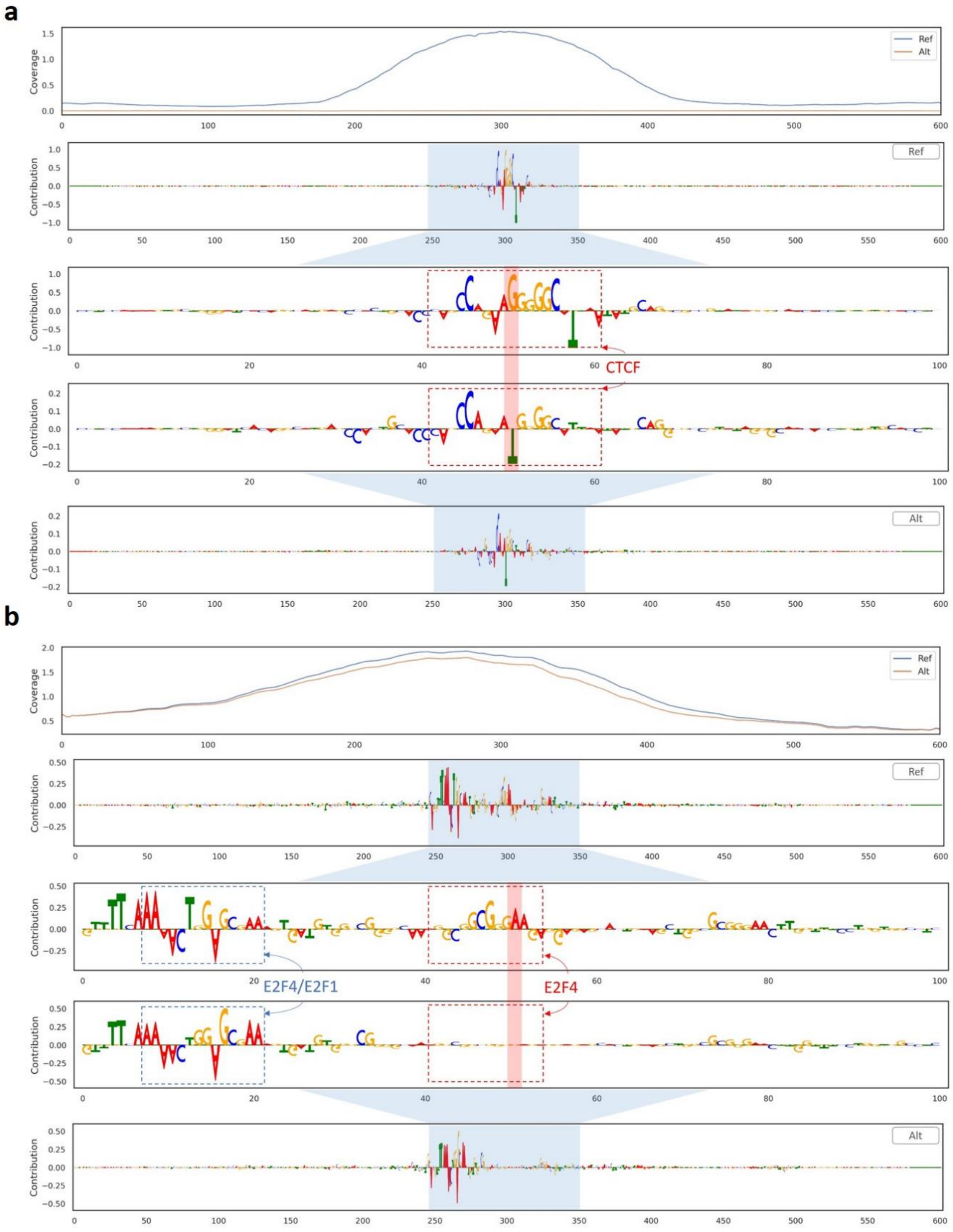

Figure S3. Interpretation of SNP. (a) Visualization of a single nucleotide variant (G→T) within CTCF binding sites, significantly affecting TF binding. (b) Visualization of a single nucleotide variant (A→G) within E2F4 binding sites, which has little effect on TF binding even though the contributions of this binding region are vanished.

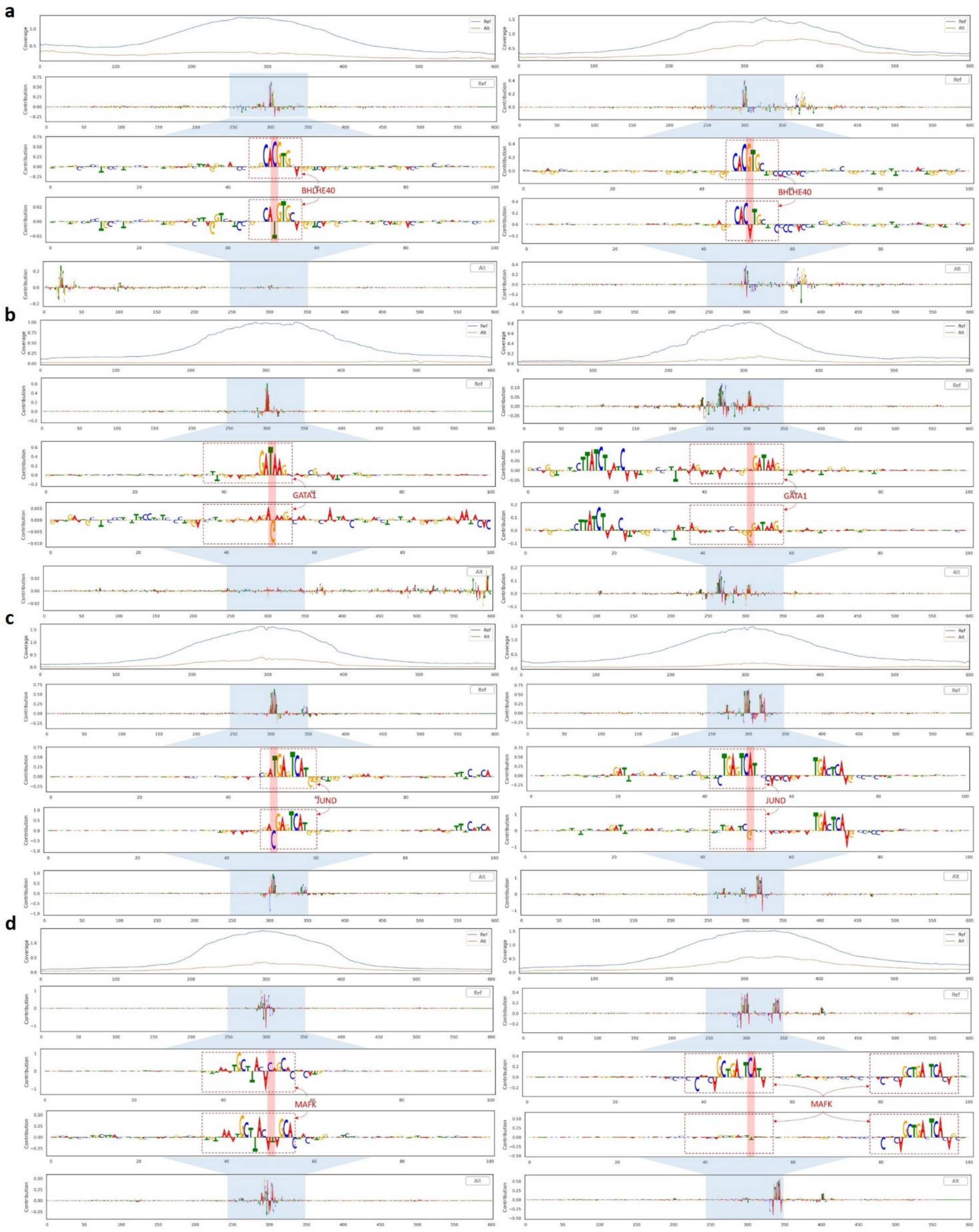

Figure S4. Examples for interpretation of SNP. (a) Visualization of single nucleotide variants (C→T, G→A) within BHLHE40 binding sites, where the first variant significantly affects TF binding while the second one transfers the contributions of TF binding prediction to its downstream cofactor. (b) Visualization of single nucleotide variants (T→G, A→G) within GATA1 binding sites, both significantly affecting TF binding. (c) Visualization of single nucleotide

variants (T→C, A→G) within JUND binding sites, both significantly affecting TF binding. (d) Visualization of single nucleotide variants (C→T, C→T) within MAFK binding sites, where the first variant significantly affects TF binding while the second one inactivates the contributions of the first MAFK binding sites to TF binding prediction (Indeed, the two binding sites jointly contribute to TF binding prediction).

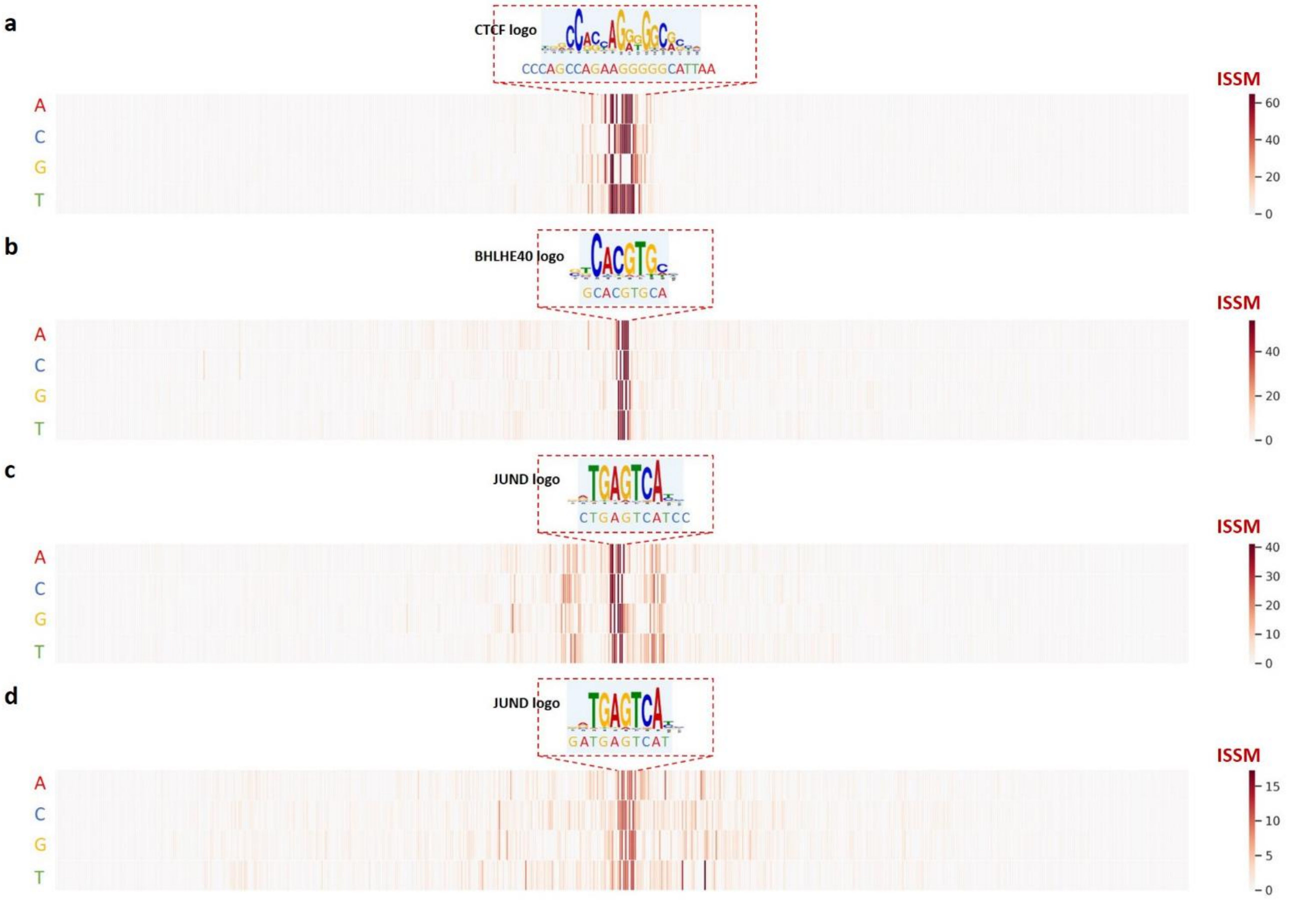

Figure S5. Examples of identifying important binding sites through ISSM. (a) Visualization of important sites of a CTCF binding sequence identified through ISSM, corresponding exactly to the CTCT motif. (b) Visualization of important sites of a BHLHE40 binding sequence identified through ISSM, corresponding exactly to the BHLHE40 motif. (c) Visualization of important sites of a JUND binding sequence identified through ISSM, corresponding exactly to the JUND motif. (d) Visualization of important sites of a JUND binding sequence identified through ISSM, corresponding exactly to the JUND motif.

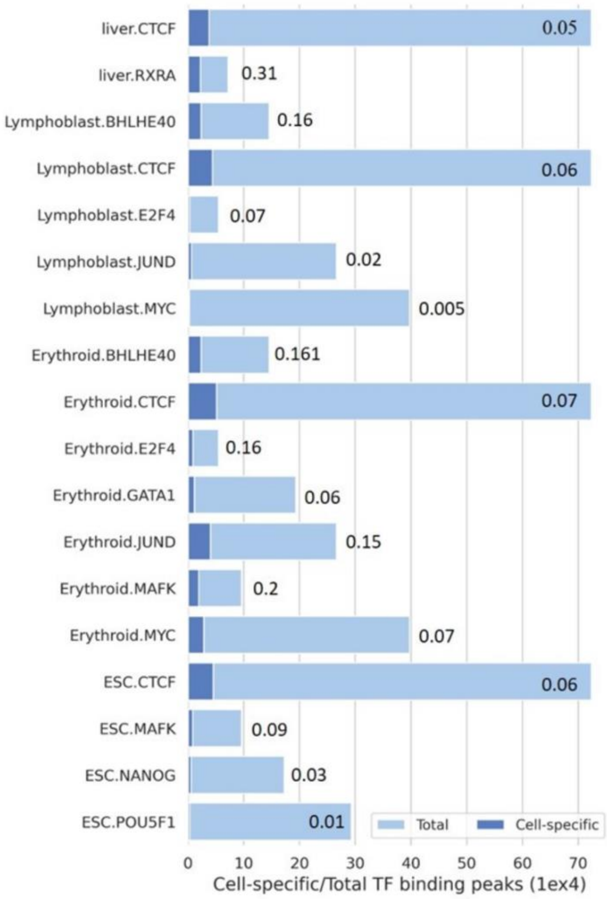

Figure S6. Proportion of cell-type-specific TF binding peaks to total peaks that are integrated from ReMap2022.

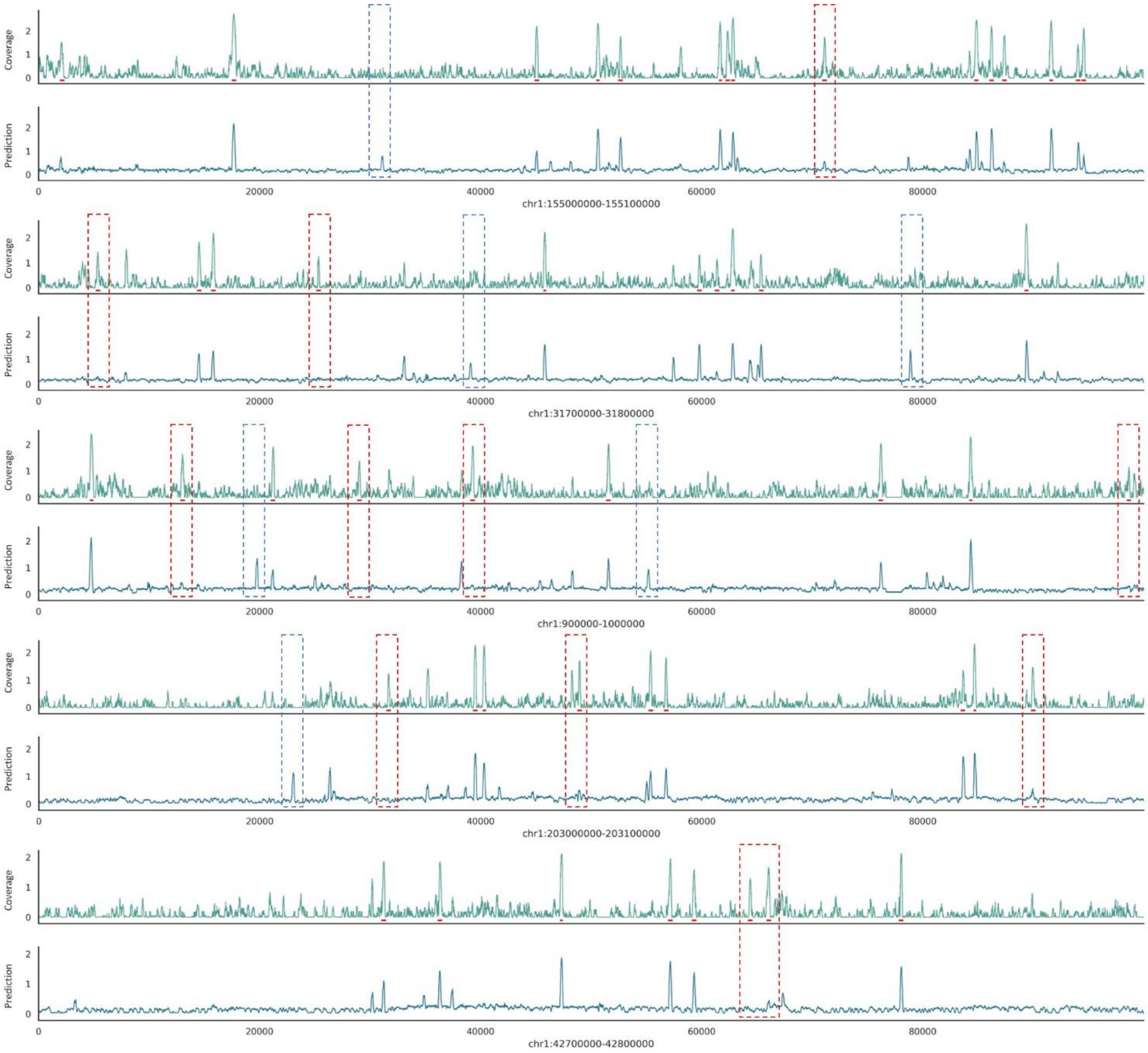

Figure S7. Visualization of true coverage values of a human genomic region (chr1:48200000-48300000, 100kb) and corresponding predicted values obtained through human, mouse, and mouse-adaptation models, where red dashed boxes represent those peaks that are mispredicted by mouse models but corrected by mouse-adaptation models.

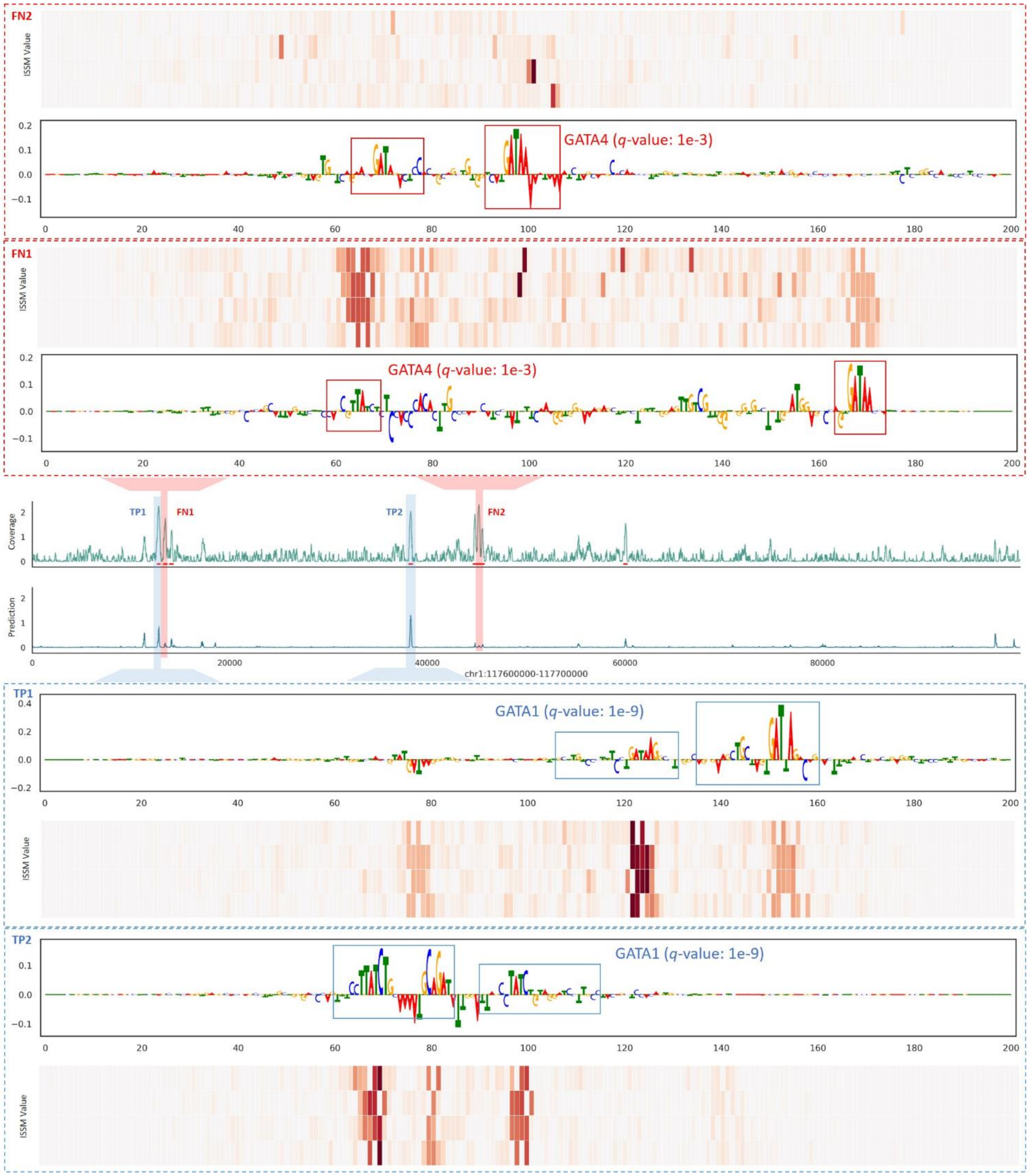

Figure S8. Visualization of some true positive (false negative) sequences from GATA1 binding regions, represented by blue (red) color, respectively.

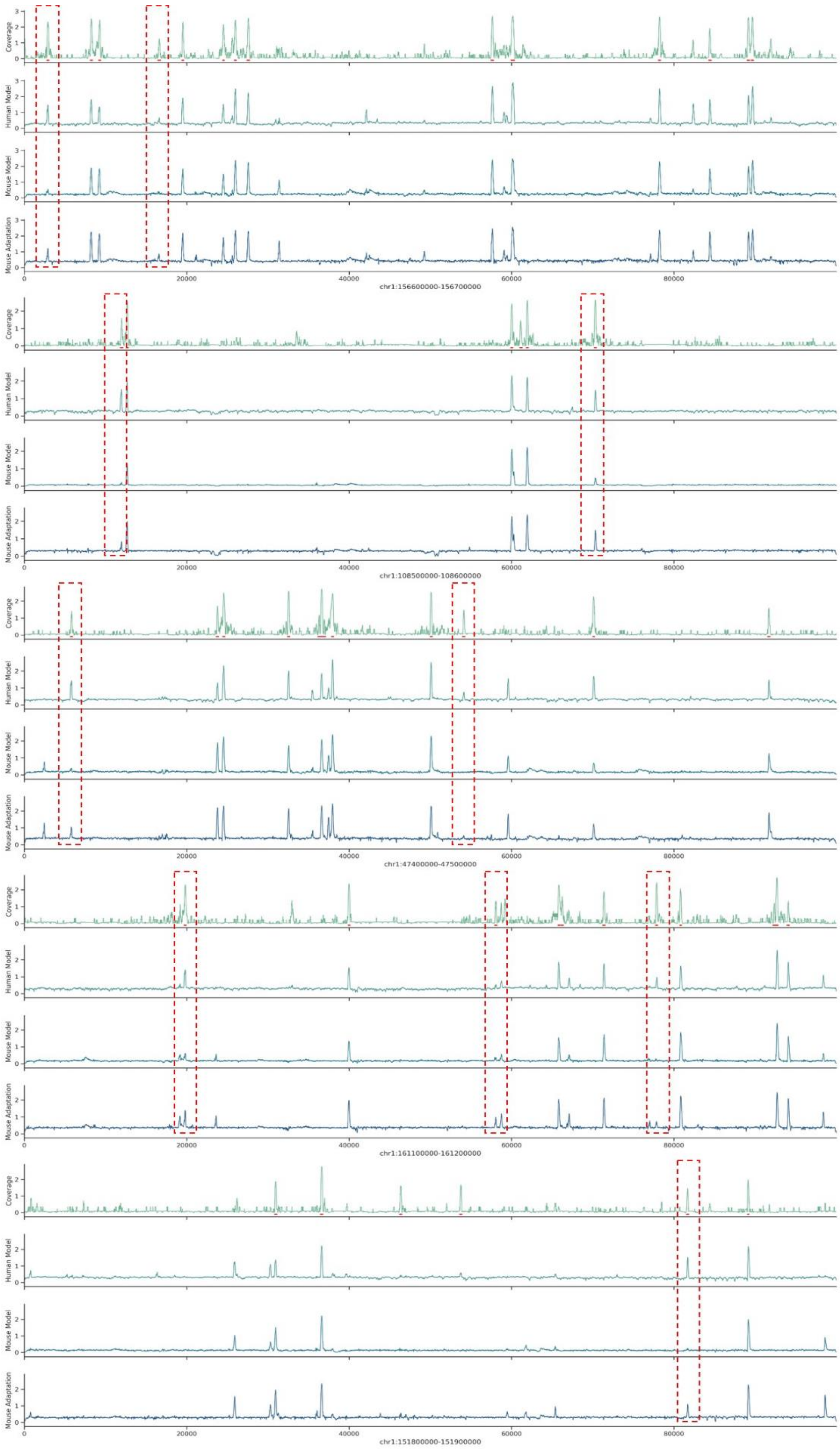

Figure S9. Visualization of true coverage values of a few human genomic regions (100kb) and corresponding predicted values obtained through human, mouse and mouse-adaptation models, where red dashed boxes represent those peaks that are mispredicted by mouse models but corrected by mouse-adaptation models.

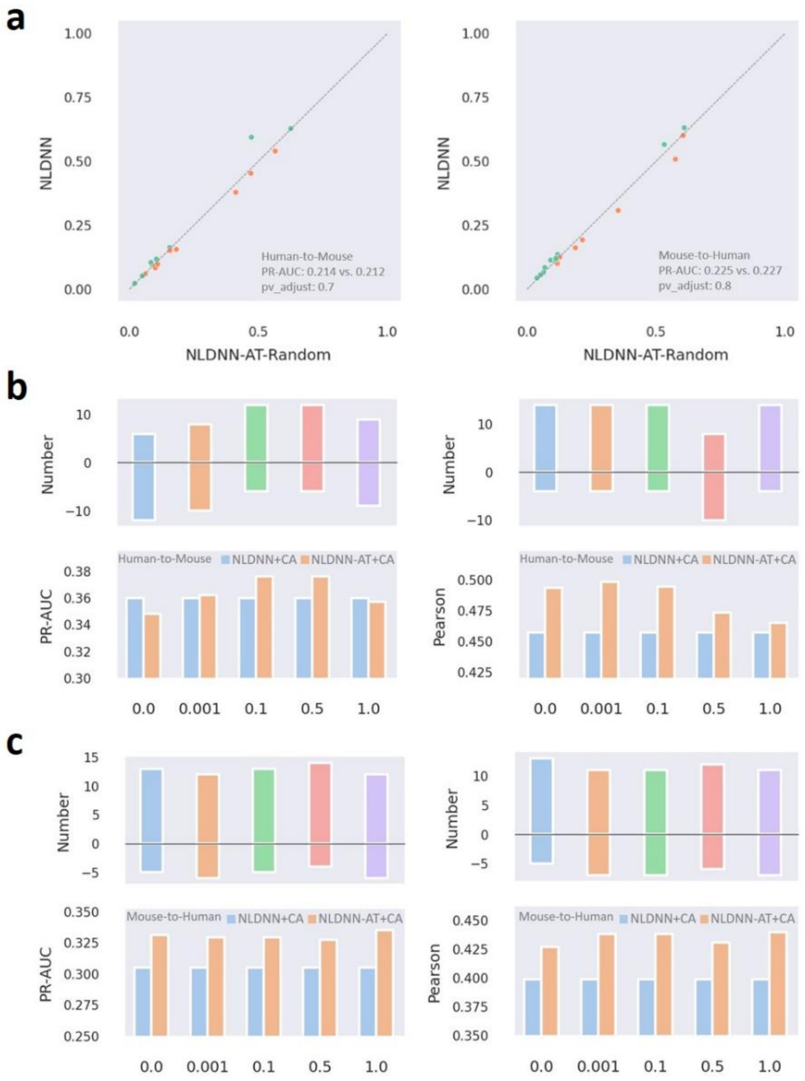

Figure S10. (a) Cross-species performance comparison between NLDNN-AT-Random and NLDNN (*p*-value is calculated using T-test, one-tailed, paired). (b) Cross-species predictive performance (Human-to-Mouse) of NLDNN-AT+CA under different proportions of binding sequences from the mouse species. (c) Cross-species predictive performance (Mouse-to-Human) of NLDNN-AT+CA under different proportions of binding sequences from the human species.

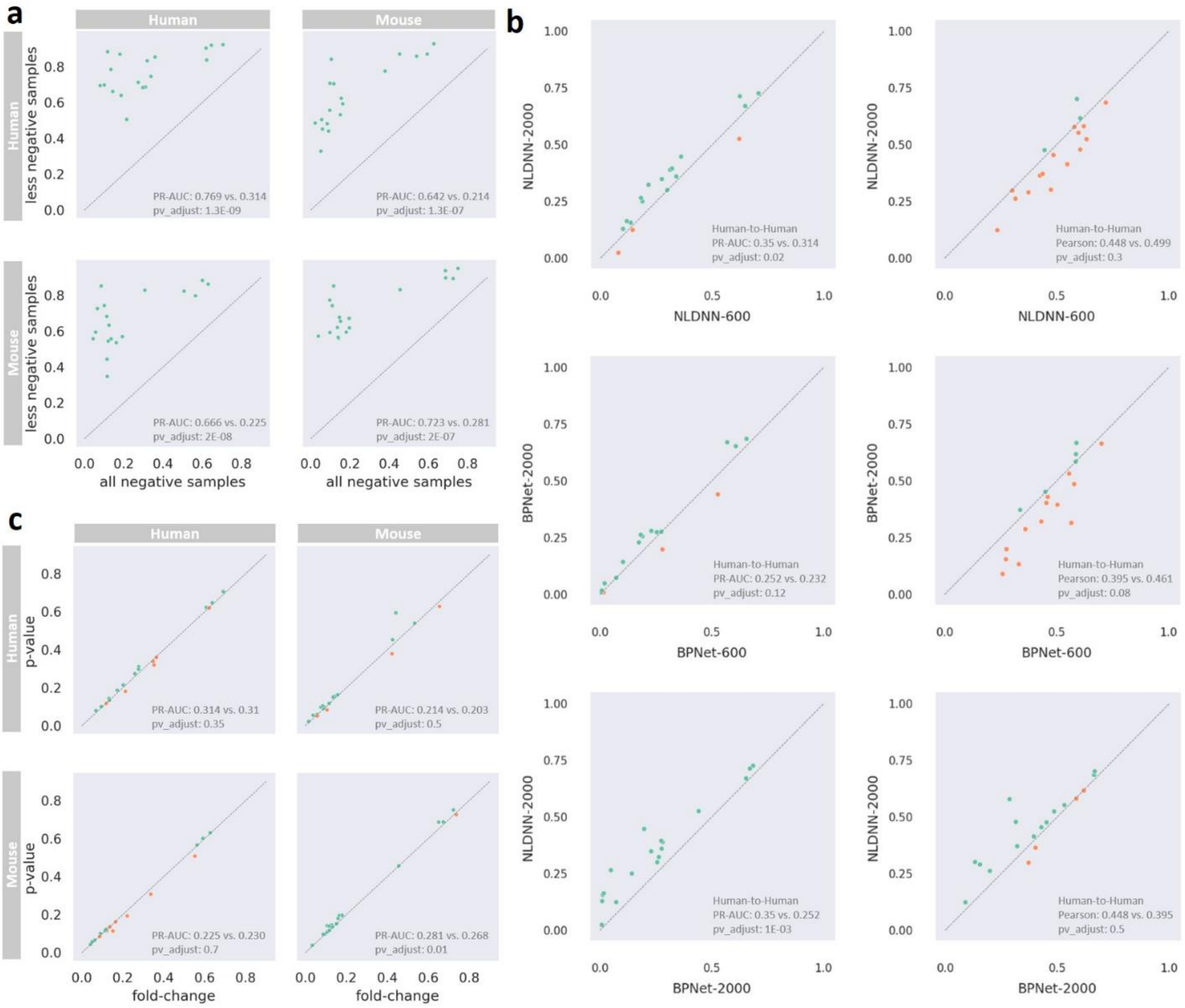

Figure S11. (a) Within- and cross-species performance comparison of NLDNN for predicting TF binding when using all or less negative samples. (b) Performance comparison of models for predicting TF binding when using DNA sequences of length 600bp or 2000bp. (c) Within- and cross-species performance comparison of NLDNN for predicting TF binding when using p-value or fold-change coverage values.

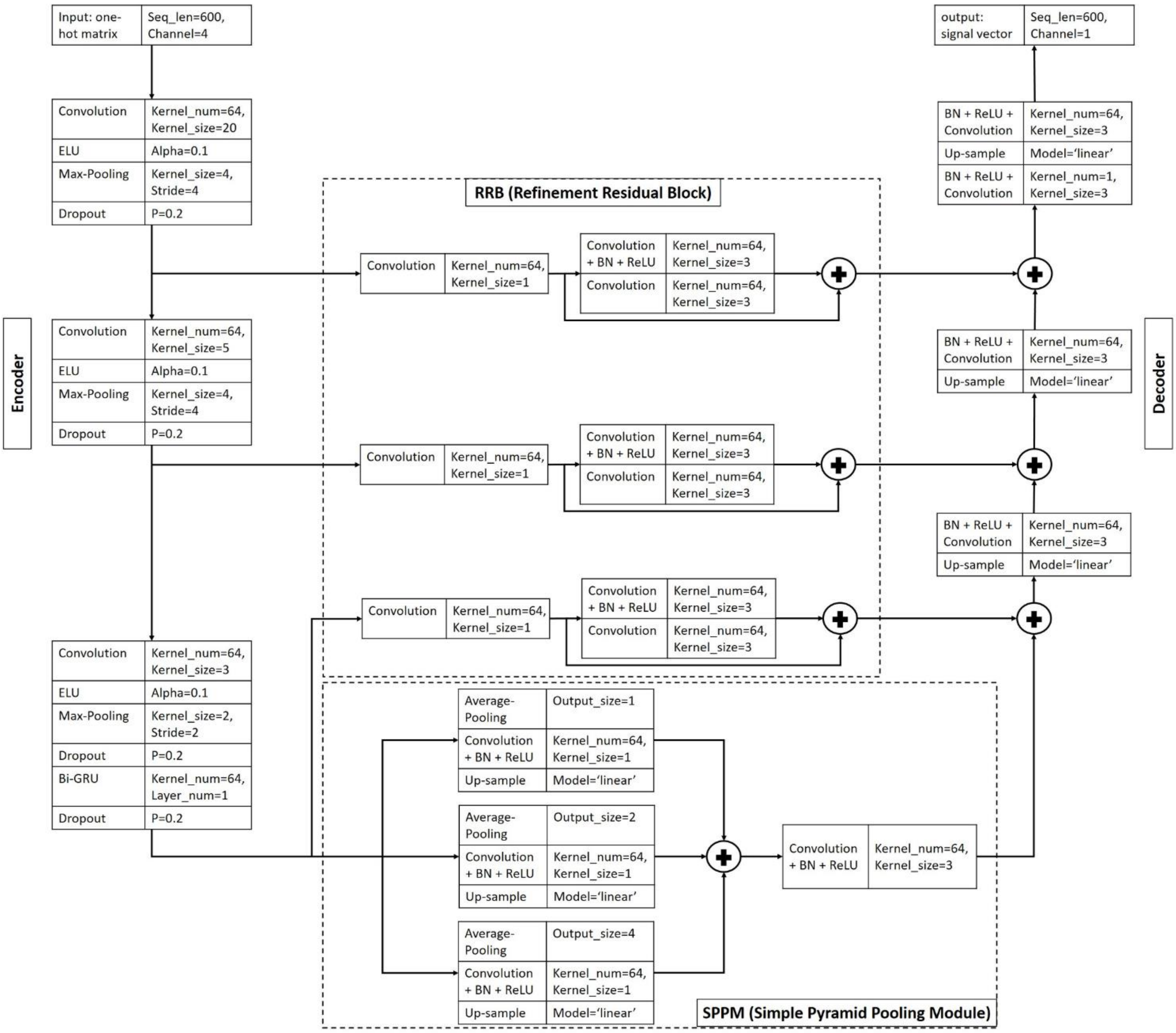

Figure S12. Detailed parameter settings of NLDNN, including Encoder, Decoder, SPPM, and RRB.

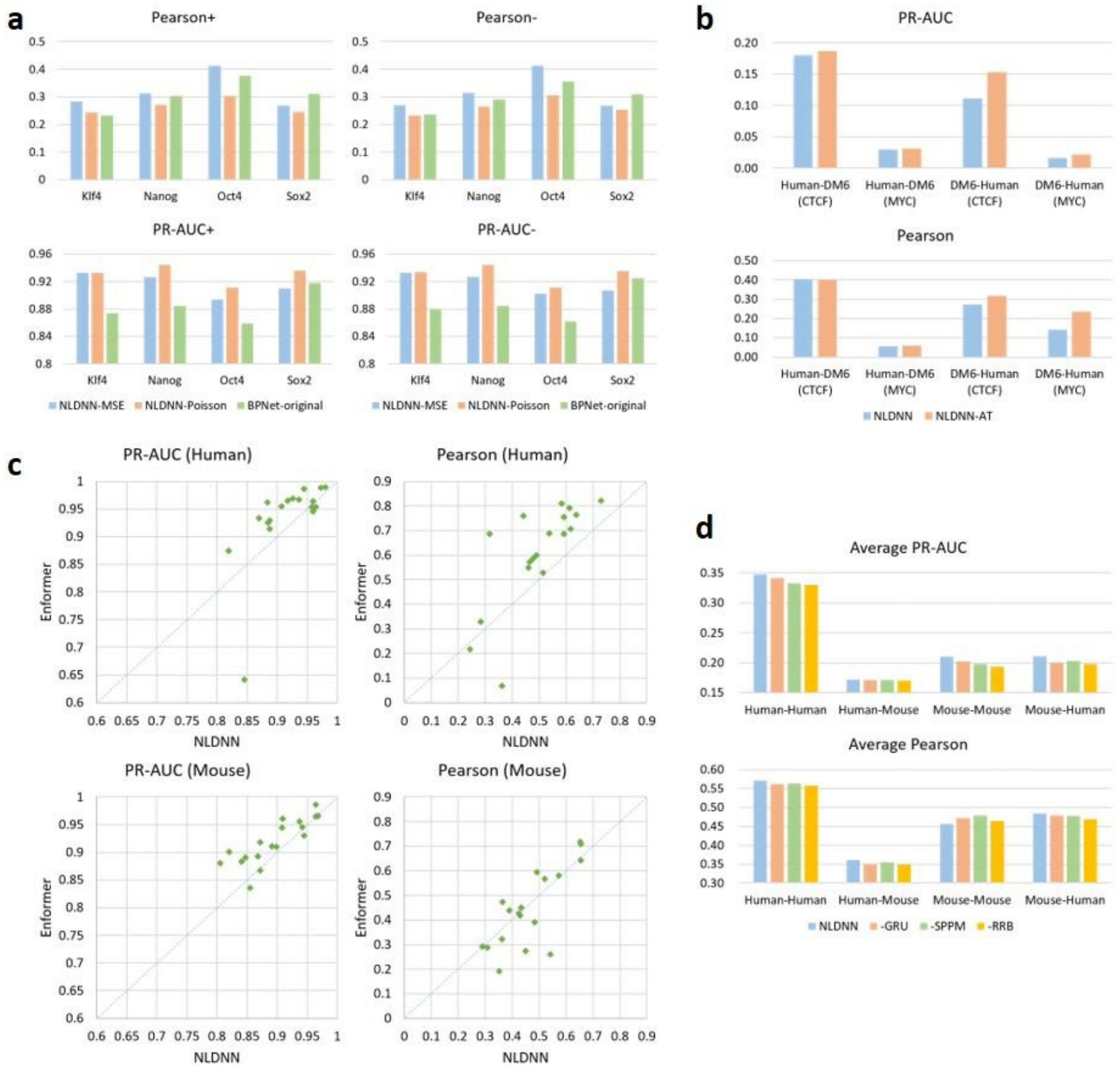

v

Figure S13. (a) The predictive performance comparison of NLDNN-MSE, NLDNN-Poisson, and BPNet-original across four Mouse ChIP-nexus binding data. (b) The cross-species predictive performance of NLDNN-AT on two ChIP-seq binding data (CTCF, MYC) from the Human and Drosophila Melanogaster species. (c) The comparison of NLDNN and Enformer for predicting within-species TF binding. (d) The predictive performance (average PR-AUC and Pearson) of ablations experiments of NLDNN

Supplementary Tables

Table S1. The accession list of ChIP-seq datasets for 18 cell-type-specific TF binding factors from human and mouse species. For coverage track files (in bigwig format) not provided by ENCODE, we used the ChIP-seq pipeline with default settings to generate corresponding coverage tracks. If there exist multiple coverage tracks for a cell type, we applied bigWigMerge and bedGraphToBigWig software from UCSC to merge them. (Note: Ratio = No. of positive sequences / No. of negative sequences.)

	**Human**			**Mouse**		
**Cell.TF**	**Peak**	**Coverage**	**Ratio**	**Peak**	**Coverage**	**Ratio**
**Ery.BHLHE40**	ENCFF242BDT	ENCFF110ZZE	7.8E-03	ENCFF142EEN	ENCFF414CZA	2.8E-03
**Ery.CTCF**	ENCFF871HJT	ENCFF268ZPN	1.0E-02	ENCFF393REY	ENCFF852JDS	6.8E-03
**Ery.E2F4**	ENCFF048ZIY	ENCFF172BRS	3.4E-03	ENCFF350EXY	ENCFF927MMJ	9.0E-04
**Ery.GATA1**	ENCFF221OWW	ENCFF331URE	3.6E-03	ENCFF024CYR	ENCFF222HZM	8.8E-03
**Ery.JUND**	ENCFF471YUP	ENCFF253NTK	1.2E-02	ENCFF744FQG	ENCFF886PYK	1.2E-03
**Ery.MAFK**	ENCFF188OEK	ENCFF408KTS	6.0E-03	ENCFF032BCM	ENCFF859RPY	1.4E-03
**Ery.MYC**	ENCFF664GSV	ENCFF140BXD	9.4E-03	ENCFF545TFJ	ENCFF944GOL	5.7E-03
**ESC.CTCF**	ENCFF497ZGV	ENCFF041TAK	8.8E-03	ENCFF052KVO	ENCFF989YLQ	8.5E-03
**ESC.MAFK**	ENCFF711HUJ	ENCFF959KBD	2.0E-03	ENCFF467JZV	ENCFF067OWO	3.9E-03
**ESC.NANOG**	ENCFF655XZF	ENCFF926YZX	1.4E-03	ENCFF453HWF	ENCFF608GQG	2.5E-03
**ESC.POU5F1**	ENCFF870LRX	ENCFF003PEE	8.0E-04	ENCFF220HTM	ENCFF771NXF	6.5E-04
**Lym.BHLHE40**	ENCFF620MJF	ENCFF325FDI	6.0E-03	ENCFF990PGN	ENCFF229EVK	8.2E-03
**Lym.CTCF**	ENCFF796WRU	ENCFF637RGD	8.0E-03	ENCFF494WBT	ENCFF255NVD	1.2E-02
**Lym.E2F4**	ENCFF629TVV	ENCFF337MYH	1.1E-03	ENCFF356FSG	ENCFF244CAE	2.5E-04
**Lym.JUND**	ENCFF825BIG	ENCFF948QYK	1.7E-03	ENCFF204CSB	ENCFF084GYC	4.2E-03
**Lym.MYC**	ENCFF765CKK	ENCFF829AJS	8.0E-04	ENCFF512CZX	ENCFF901VFB	6.3E-03

**liver.CTCF**	GSE105829	/	9.6E-03	ENCSR000CBU	ENCFF155SPJ	6.0E-03
**liver.RXRA**	ENCSR098XMN	ENCFF301XZS	1.9E-02	GSM1299600	/	6.0E-03
	# Chromation accessibility (Coverage tracks)					
**Cell**	**Human (Coverage)**			**Mouse (Coverage)**		
**Erythroid**	ENCFF600FDO			ENCFF145VWQ; ENCFF630ILP; ENCFF359CLY		
**ESC**	ENCFF232GUZ; ENCFF492ENM			ENCFF672DJH; ENCFF962TCT		
**Lymphoblast**	ENCFF180ZAY			ENCFF228LWM; ENCFF315JOG		
**liver**	ENCFF435JGG			ENCFF982MGZ; ENCFF947ZHU		

Table S2. The accession list of chromatin accessibility from different cell lines.

Cell	Human (Peak)	Human (Coverage)
**HepG2**	ENCFF438JMM	ENCFF285FQS
**Panc1**	ENCFF481ELM	ENCFF794CNJ
**HEK293T**	ENCFF285OXK	ENCFF529BOG
**SK-MEL-5**	ENCFF659HYC	ENCFF627WEJ
**K562**	ENCFF925CYR	ENCFF600FDO
**HeLa-S3**	ENCFF049BHH	ENCFF757GHL
**GM12878**	ENCFF945SYZ	ENCFF180ZAY
**NHEK**	ENCFF245IFS	ENCFF839EGC

Table S3. A complementary comparison of all methods, focusing on computational efficiency, and interpretability. Inference time was obtained by calculating the average time for 1000 runs.

**Method**	**Type**	**No. of Flops**	**No. of Params**	**Inference Time (ms)**	**Interpretability**	**Year**	**Availability**
DeepSea/DeepSea+	Sequence-level	6.3E+06	1.1E+05	2	No	2015	http://deepsea.princeton.edu
DanQ/DanQ+	Sequence-level	1.4E+08	3.6E+07	7	No	2016	http://github.com/uci-cbcl/DanQ
DanQV/DanQV+	Sequence-level	1.5E+07	1.9E+06	2	No	2022	https://github.com/seqcode/cross-species-domain-adaptation
FCNA	Nucleotide-level	7.1E+06	6.4E+04	4	No	2021	https://github.com/turningpoint1988/FCNA
Leopard	Nucleotide-level	3.2E+07	4.9E+05	10	No	2021	https://github.com/GuanLab/Leopard
FCNsignal	Nucleotide-level	9.8E+06	1.3E+05	9	No	2022	https://github.com/turningpoint1988/FCNsignal
BPNet	Nucleotide-level	7.3E+07	1.2E+05	12	Yes	2021	https://github.com/kundajelab/bpnet
NLDNN	Nucleotide-level	1.6E+07	2.4E+05	11	Yes	2024	https://github.com/turningpoint1988/NLDNN-AT
Enformer	Bin-level	6.2E+12	4.8E+08	23880	Yes	2021	https://github.com/google-deepmind/deepmind-research/tree/master/enformer
